# Near-Infrared Spectroscopy-Guided Selective Distal Perfusion in Femoral Venoarterial Extracorporeal Membrane Oxygenation: A Feasibility Study

**DOI:** 10.3390/diagnostics16203361

**Published:** 2026-10-15

**Authors:** Woong-Su Yoon, Yerin Kim, Ju-Seung Kwun, Ki-Hyun Jeon, Sang Hon Park, Hyoung-Won Cho

**Affiliations:** 1 Department of Internal Medicine, Chungbuk National University Hospital, Chungbuk National University College of Medicine, Cheongju 28644, Republic of Korea; yoonws0819@gmail.com; 2 Division of Cardiology, Department of Internal Medicine, Seoul National University Bundang Hospital, Seongnam 13620, Republic of Korea; yaerin9063@gmail.com (Y.K.);; 3 Department of Internal Medicine, Seoul National University College of Medicine, Seoul 03080, Republic of Korea; 4 Department of Thoracic and Cardiovascular Surgery, Seoul National University Bundang Hospital, Seongnam 13620, Republic of Korea; 5 Department of Thoracic and Cardiovascular Surgery, Seoul National University College of Medicine, Seoul 03080, Republic of Korea

**Keywords:** extracorporeal membrane oxygenation, near-infrared spectroscopy, distal perfusion catheter, limb ischemia, extracorporeal cardiopulmonary resuscitation, cardiogenic shock

## Abstract

**Background/Objectives:** We evaluated the feasibility of a near-infrared spectroscopy (NIRS)-guided selective strategy based on a single absolute regional tissue oxygen saturation (rSO_2_) threshold and described clinically overt limb ischemia. **Methods:** This single-center retrospective study included 110 adults receiving femoral venoarterial extracorporeal membrane oxygenation between April 2020 and December 2024. All underwent bilateral lower-extremity NIRS monitoring. In 83 patients, distal perfusion catheter (DPC) placement was triggered by rSO_2_ < 40% in the cannulated limb or clinically evident limb ischemia; 27 received routine prophylactic DPC placement at cannulation. Primary endpoints were DPC avoidance and clinically overt limb ischemia. **Results:** Extracorporeal cardiopulmonary resuscitation (ECPR) accounted for 64.5% of patients. Under the selective strategy, 45/83 patients (54.2%; exact 95% CI, 42.9–65.2%) avoided DPC placement, with no clinically overt limb ischemia (0/45; exact 95% CI, 0–7.9%). Limb ischemia occurred in 2/83 versus 2/27 patients (2.4% versus 7.4%; risk difference, −5.0 percentage points; 95% CI, −21.1 to +3.0; *p* = 0.252). Because threshold crossing triggered DPC placement, the reported threshold characteristics reflect the operation of the management protocol rather than independent diagnostic accuracy. **Conclusions:** A single-threshold NIRS-guided selective strategy was feasible in this ECPR-dominant population, allowing just over half of patients to avoid prophylactic DPC placement. No clinically overt limb ischemia was observed in this subgroup, but the upper 95% confidence limit of 7.9% precludes a definitive safety conclusion. Prospective studies are needed to assess limb ischemia risk and refine selection criteria.

## 1. Introduction

Venoarterial extracorporeal membrane oxygenation (VA-ECMO) is an established form of temporary mechanical circulatory support for patients with refractory cardiogenic shock and cardiac arrest [1,2]. Peripheral cannulation through the femoral vessels remains the most commonly used configuration because of its rapid and relatively straightforward percutaneous insertion, particularly in emergency settings such as extracorporeal cardiopulmonary resuscitation (ECPR) [3].

Femoral arterial cannulation, however, can compromise antegrade blood flow to the ipsilateral lower extremity and result in limb ischemia, with reported incidence varying substantially according to patient population, definition, and monitoring strategy [4,5,6,7]. Severe limb ischemia may require surgical intervention, fasciotomy, or amputation and can complicate an already critical clinical course [6,7]. A distal perfusion catheter (DPC), typically inserted into the superficial femoral artery to provide antegrade distal flow, is widely used to mitigate this risk, and prophylactic DPC placement has been associated with lower rates of limb ischemia [8,9,10,11].

Routine prophylactic DPC placement, however, exposes all patients to an additional invasive vascular procedure and may itself be associated with bleeding, thrombosis, vascular injury, pseudoaneurysm, or infection [3,12]. Because not all patients develop distal limb hypoperfusion during VA-ECMO support, a selective strategy that identifies patients in whom DPC placement can be deferred may reduce procedural burden while maintaining adequate surveillance for limb ischemia.

Near-infrared spectroscopy (NIRS) provides continuous, noninvasive assessment of regional tissue oxygen saturation (rSO_2_) and is particularly attractive during VA-ECMO because many patients are sedated, comatose, or otherwise unable to report symptoms of limb ischemia [5,13,14]. Previous studies have demonstrated the feasibility of lower-extremity NIRS for detecting hypoperfusion and guiding distal perfusion management, and recent evidence has further supported NIRS-based limb surveillance and selective DPC strategies [4,15,16,17]. However, criteria for selective DPC placement remain heterogeneous, often incorporating absolute rSO_2_ values, changes from baseline, or inter-limb differences. In emergency cannulation, particularly during ECPR or profound cardiogenic shock, reliable pre-cannulation baseline measurements may not be available, limiting the practical application of relative-change criteria.

We therefore evaluated a NIRS-guided selective distal perfusion strategy using a single absolute rSO_2_ threshold in adults undergoing femoral VA-ECMO. The primary aim was to assess the feasibility of avoiding prophylactic DPC placement and to describe clinically overt limb ischemia under this strategy. As a secondary exploratory analysis, outcomes were compared with those of patients who underwent routine prophylactic DPC placement during the same study period.

## 2. Materials and Methods

### 2.1. Study Design and Population

This single-center retrospective feasibility study evaluated a NIRS-guided selective distal perfusion strategy in adult patients who underwent peripheral VA-ECMO with femoral arterial cannulation at Seoul National University Bundang Hospital between April 2020 and December 2024. Of 116 consecutive patients identified from the institutional ECMO registry, 6 were excluded: 2 without femoral arterial return cannulation (1 with axillary arterial cannulation and 1 converted from a temporary left ventricular assist device), 2 in whom ECMO support could not be established, 1 patient younger than 18 years, and 1 patient cannulated at a referring hospital before transfer, in whom the institutional distal perfusion protocol could not be applied from the time of cannulation. The final analytic cohort comprised 110 patients.

Distal perfusion strategy was determined at the time of ECMO cannulation according to the preference of the implanting physician; no written institutional criterion governed this choice. In total, 83 patients were managed using a NIRS-guided selective DPC strategy, whereas 27 underwent routine prophylactic DPC placement at the time of ECMO cannulation. Both strategies were used throughout the study period rather than sequentially (routine prophylactic DPC, 7–36% of cannulations per year; Cochran–Armitage test for trend, *p* = 0.136). Bilateral lower-extremity NIRS monitoring was performed in both strategies; however, only in the selective strategy were rSO_2_ values used to guide subsequent DPC placement. All patients presented with cardiogenic shock or cardiac arrest requiring mechanical circulatory support.

### 2.2. Distal Perfusion Strategies and Patient Classification

Patients were classified according to the distal perfusion strategy selected at the time of ECMO cannulation.

NIRS-guided selective DPC strategy (*n* = 83). Patients underwent bilateral lower-extremity NIRS monitoring without routine DPC placement at the time of ECMO cannulation. Subsequent DPC placement was performed when the protocol-defined rSO_2_ trigger was reached or when clinically evident limb ischemia developed. For descriptive analyses, patients in this strategy were further classified according to whether a DPC was ultimately placed during ECMO support: the no-DPC subgroup (*n* = 45), comprising patients who did not receive a DPC during ECMO support, and the selective DPC subgroup (*n* = 38), comprising patients who underwent DPC placement after cannulation because the trigger was reached or clinically evident limb ischemia developed.

Routine prophylactic DPC strategy (*n* = 27). Patients underwent DPC placement at the time of ECMO cannulation according to the preference of the implanting physician. Bilateral lower-extremity NIRS monitoring was also performed in this group; however, rSO_2_ values were not used to determine whether a DPC should be placed, because prophylactic DPC insertion had already been performed at cannulation.

### 2.3. ECMO Cannulation and NIRS Monitoring

Peripheral VA-ECMO was established using percutaneous femoral cannulation under fluoroscopic or ultrasound guidance when available. Arterial return cannulae ranged from 15 to 19 Fr (15 Fr, *n* = 73; 17 Fr, *n* = 29; 19 Fr, *n* = 3; not recorded, *n* = 5), and venous drainage cannulae ranged from 21 to 24 Fr. Unfractionated heparin was the primary anticoagulant (104/110, 94.5%). Both strategies were used during the same study period by the same institutional ECMO team.

Bilateral lower-extremity NIRS sensors were placed over the posterior calf overlying the gastrocnemius muscle at the time of ECMO cannulation using the INVOS 5100C system (Somanetics, Troy, MI, USA). rSO_2_ was monitored continuously throughout VA-ECMO support and for 24 h after ECMO weaning in both distal perfusion strategies.

In the selective strategy, the decision to place a DPC was based on the absolute rSO_2_ value of the cannulated limb. An rSO_2_ < 40% or clinically evident limb ischemia triggered DPC insertion. No inter-limb difference criterion or relative decline from a pre-cannulation baseline was used for DPC decision-making.

A universally accepted normal range for lower-extremity rSO_2_ has not been established; absolute values depend on measurement conditions and systemic perfusion. Previous ECMO studies have used different criteria for lower-extremity hypoperfusion, including absolute thresholds, changes from baseline, and inter-limb differences [5,15,17]. The 40% threshold in this protocol was informed by prior reports and chosen as a pragmatic bedside trigger during emergency VA-ECMO cannulation, when reliable baseline measurements are often unavailable. The 40% threshold was used as a pragmatic, protocol-defined intervention threshold rather than as a validated diagnostic cutoff. An rSO_2_ value at or above 40% should therefore not be interpreted as a universal normal value or as excluding limb ischemia. Severe systemic hypoperfusion and vasopressor use may affect tissue oxygenation in both lower extremities, limiting the reliability of the contralateral limb as an internal reference.

### 2.4. Outcomes

The primary feasibility endpoint was the proportion of patients managed under the NIRS-guided selective strategy who did not receive a DPC during ECMO support. The primary safety endpoint was clinically overt limb ischemia of the cannulated extremity.

Because many patients were sedated or comatose, limb ischemia was defined using objective clinical findings that did not require patient cooperation, including new pallor, mottling, or coldness of the cannulated limb, loss of distal arterial Doppler signals, or compartment syndrome. Therapeutic interventions for established limb ischemia included DPC placement, thrombectomy, fasciotomy, or amputation. DPC placement based solely on the protocol-defined rSO_2_ trigger, in the absence of clinically documented ischemia, was not considered a limb ischemia event. Vascular events that were clinically determined to be unrelated to ECMO cannulation or distal limb hypoperfusion were not classified as ECMO-related limb ischemia.

ECMO-related complications were defined as cannula-, vascular-, or circuit-related complications, including limb ischemia, arterial dissection or injury, arteriovenous fistula, cannula-site hematoma or bleeding, cannula malposition, and circuit malfunction. Secondary outcomes included in-hospital mortality (all-cause and cardiac death), favorable neurological outcome defined as a Cerebral Performance Category (CPC) score of 1–2 at discharge, requirement for continuous renal replacement therapy (CRRT), sepsis, bleeding, ECMO support duration, and length of hospital stay.

### 2.5. Descriptive Analysis of Limb Ischemia by Distal Perfusion Status

Within the NIRS-guided selective strategy cohort, limb ischemia was cross-tabulated according to whether a DPC was subsequently placed (selective DPC vs. no DPC). Because the same rSO_2_ decline that triggered DPC placement also determined membership in the selective DPC subgroup, and DPC placement itself could modify the subsequent risk of limb ischemia, these analyses were considered descriptive. Event rates with exact 95% confidence intervals were therefore emphasized. Conventional test characteristics of the rSO_2_ threshold are reported as descriptive measures rather than as estimates of independent diagnostic accuracy.

### 2.6. Statistical Analysis

The primary comparative analysis was performed according to the distal perfusion strategy selected at the time of ECMO cannulation: NIRS-guided selective DPC versus routine prophylactic DPC. Continuous variables are presented as median (interquartile range [IQR]) and were compared using the Mann–Whitney U test. Categorical variables are presented as number (percentage) and were compared using the chi-square test or Fisher’s exact test, as appropriate. Absolute risk differences are reported with Newcombe hybrid score 95% confidence intervals, and proportions with Clopper–Pearson exact 95% confidence intervals.

Because distal perfusion strategy was selected according to operator preference rather than random allocation, baseline characteristics between the two strategies were additionally assessed using standardized mean differences (SMDs). Given the limited sample size and small number of limb ischemic events, no propensity-score adjustment or multivariable modeling of limb ischemia was performed.

A descriptive three-group analysis according to eventual DPC status (no DPC, selective DPC, and routine prophylactic DPC) was performed using the Kruskal–Wallis test for continuous variables and the chi-square or Fisher–Freeman–Halton exact test for categorical variables, as appropriate, and is provided in the Supplementary Materials.

ECMO support duration was calculated from cannulation to decannulation. When the decannulation date was unavailable, the date of heart transplantation or in-hospital death was used as the end of ECMO support; the date of in-hospital death was derived as the admission date plus the recorded length of stay, with the day of admission counted as day 1. A two-sided *p* value < 0.05 was considered statistically significant. All analyses were performed using Python 3.11 with SciPy 1.17 and statsmodels 0.15 (Python Software Foundation, Wilmington, DE, USA).

## 3. Results

### 3.1. Study Population

**Figure 1:**
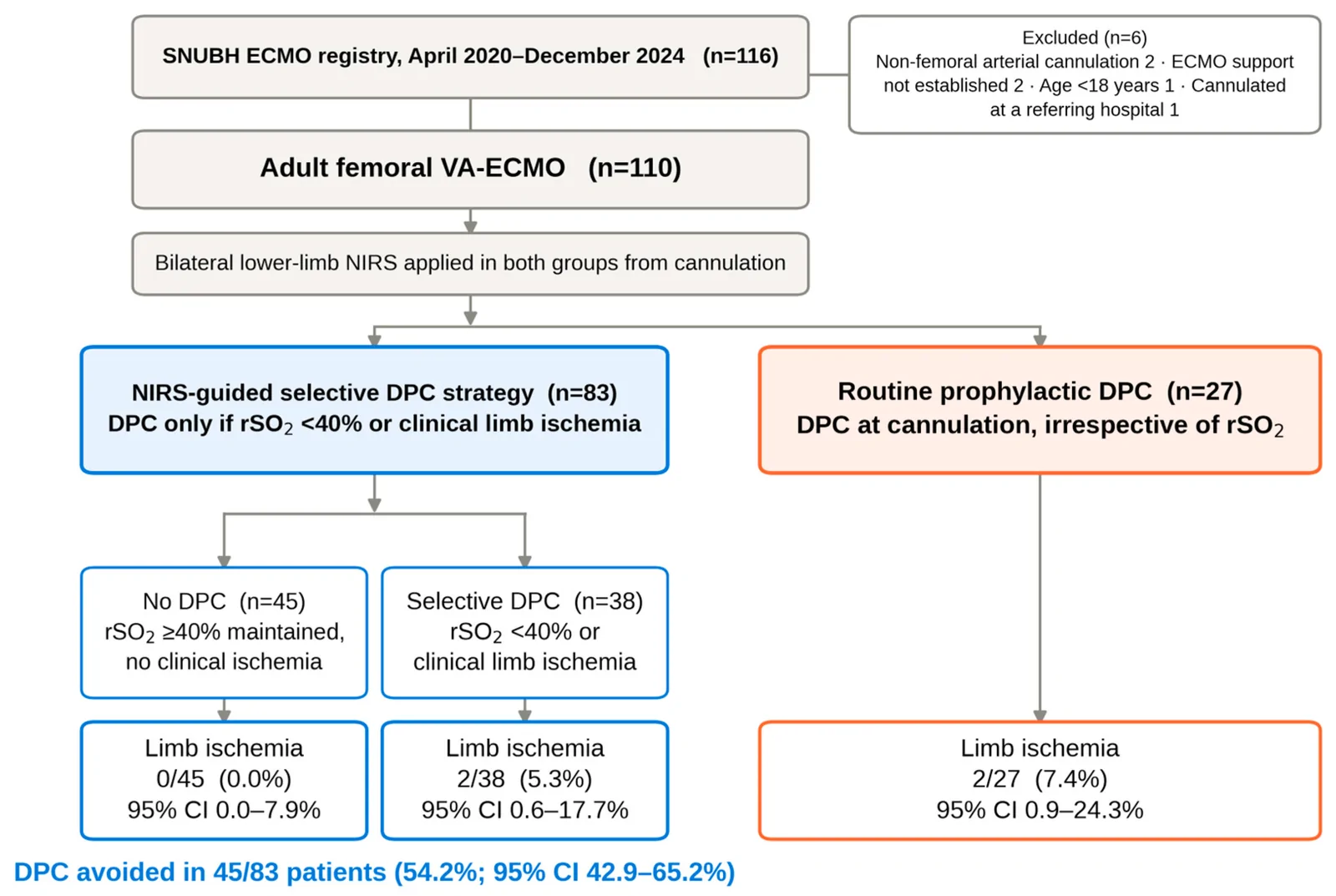
Study flow and clinically overt limb ischemia according to distal perfusion strategy. Of 116 consecutive patients in the institutional ECMO registry, 6 were excluded, leaving 110 adults supported with femoral VA-ECMO. Bilateral lower-extremity NIRS monitoring was performed in both strategies; only in the NIRS-guided selective strategy (*n* = 83) were rSO_2_ values used to guide DPC placement. DPC, distal perfusion catheter; NIRS, near-infrared spectroscopy; rSO_2_, regional tissue oxygen saturation; VA-ECMO, venoarterial extracorporeal membrane oxygenation.

Of the 110 patients included in the analysis, 83 were managed using the NIRS-guided selective DPC strategy and 27 underwent routine prophylactic DPC placement at the time of ECMO cannulation (Figure 1). The median age was 66.0 years (IQR, 52.0–74.8), 70.9% were male, and 64.5% underwent ECPR.

Baseline characteristics according to distal perfusion strategy are shown in Table 1. Although conventional between-group comparisons were not statistically significant for age (66.0 (52.0–74.5) vs. 65.0 (53.5–75.0) years; *p* = 0.602), ECPR (68.7% vs. 51.9%; *p* = 0.112), SOFA score (10.0 (8.0–13.0) vs. 9.5 (6.8–12.0); *p* = 0.227), arterial cannula size (*p* = 0.677), or anticoagulation with unfractionated heparin (*p* = 0.333), the SMDs indicated imbalance in several characteristics, including SOFA score (0.40), ECPR (0.35), and unfractionated heparin use (−0.39). Fluoroscopic guidance showed the largest imbalance and was more frequently used in the routine prophylactic DPC group (66.7% vs. 28.9%; *p* < 0.001; SMD, −0.82). Median ECMO support duration was 6 (3–10) days in the selective strategy group and 8 (6–16) days in the routine prophylactic DPC group (*p* = 0.024).

### 3.2. Primary Feasibility Outcome: DPC Avoidance

Among the 83 patients managed under the NIRS-guided selective strategy, 45 did not receive a DPC during ECMO support, corresponding to a DPC avoidance rate of 54.2% (exact 95% CI, 42.9–65.2%). The remaining 38 patients (45.8%) underwent selective DPC placement after ECMO cannulation because the protocol-defined rSO_2_ trigger was reached or clinically evident limb ischemia developed.

### 3.3. Primary Safety Outcome: Limb Ischemia

Clinically overt limb ischemia occurred in 2 of 83 patients managed under the NIRS-guided selective strategy (2.4%; exact 95% CI, 0.3–8.4%) and in 2 of 27 patients who underwent routine prophylactic DPC placement (7.4%; exact 95% CI, 0.9–24.3%). The absolute risk difference was −5.0 percentage points (95% CI, −21.1 to +3.0; *p* = 0.252).

Within the NIRS-guided selective strategy cohort, both limb ischemia events occurred among patients who subsequently received a selective DPC (2/38, 5.3%; exact 95% CI, 0.6–17.7%). No clinically overt limb ischemia was observed among the 45 patients managed without a DPC (0/45; exact 95% CI, 0–7.9%).

The four limb ischemia events are summarized in Table S1. In both patients who developed clinically overt limb ischemia under the selective strategy, DPC placement followed an rSO_2_ decline, and clinically overt limb ischemia was reported after DPC placement. The recorded rSO_2_ at the DPC trigger was <40% in one patient and 34% in the other. Precise intervals between the rSO_2_ decline, DPC insertion, and recognition of clinically overt ischemia were not systematically available. The first patient had catecholamine-induced cardiogenic shock; the second had Takayasu arteritis with previous ECMO support. Both underwent fasciotomy, were successfully weaned from ECMO, and survived to hospital discharge with a CPC score of 1. Both limb ischemia events in the routine prophylactic DPC group occurred despite DPC placement at the time of cannulation, and both patients died during hospitalization.

Cannula-, vascular-, or circuit-related complications occurred in 12 of 83 patients managed under the selective strategy (14.5%) and in 2 of 27 patients in the routine prophylactic DPC group (7.4%; *p* = 0.511). These events included limb ischemia, femoral arterial dissection or injury, arteriovenous fistula, cannula-site bleeding or hematoma, and circuit-related complications.

### 3.4. Limb Ischemia According to Distal Perfusion Status

**Figure 2:**
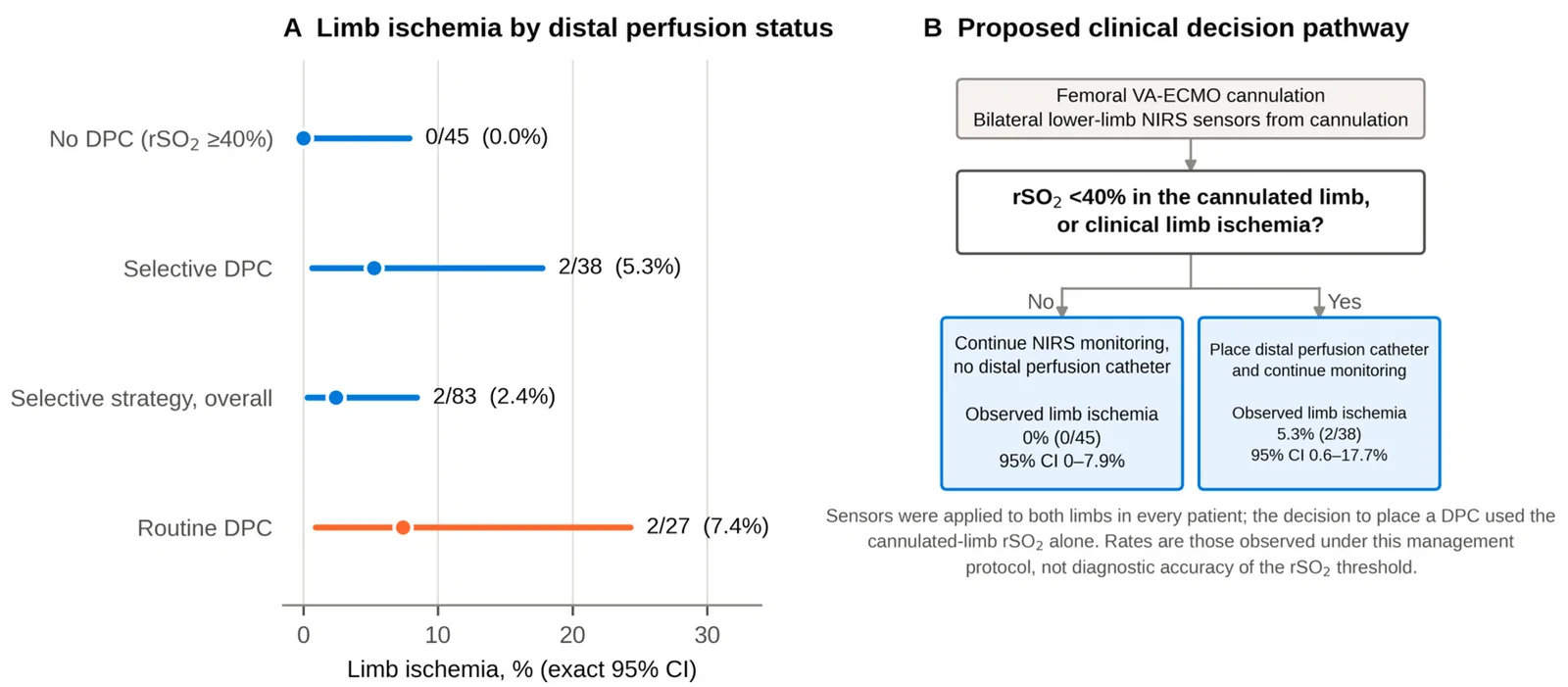
Clinically overt limb ischemia according to distal perfusion status and the corresponding decision pathway. (**A**) Observed limb ischemia with Clopper–Pearson exact 95% confidence intervals in the no-DPC and selective DPC subgroups, in the selective strategy overall, and in the routine prophylactic DPC group. In panel A, blue denotes the NIRS-guided selective strategy and its subgroups, and orange denotes the routine prophylactic DPC group. (**B**) Decision pathway applied under the NIRS-guided selective strategy, annotated with the limb ischemia rate observed in each branch. Sensors were applied to both limbs in every patient, but the decision to place a DPC used the absolute rSO_2_ value of the cannulated limb alone; the rates shown are those observed within this management protocol and do not represent diagnostic accuracy of the threshold. DPC, distal perfusion catheter; NIRS, near-infrared spectroscopy; rSO_2_, regional tissue oxygen saturation; VA-ECMO, venoarterial extracorporeal membrane oxygenation.

Within the NIRS-guided selective strategy cohort, limb ischemia occurred in 2 of 38 patients who subsequently received a selective DPC (5.3%; exact 95% CI, 0.6–17.7%) and in none of the 45 patients managed without a DPC (0%; exact 95% CI, 0–7.9%; Fisher’s exact *p* = 0.207) (Figure 2A).

For descriptive purposes, the operational characteristics of the protocol-defined rSO_2_ threshold of <40% in relation to subsequent clinically overt limb ischemia were as follows: sensitivity, 100% (2/2; exact 95% CI, 15.8–100.0%); specificity, 55.6% (45/81; exact 95% CI, 44.1–66.6%); positive predictive value, 5.3% (2/38; exact 95% CI, 0.6–17.7%); and negative predictive value, 100% (45/45; exact 95% CI, 92.1–100.0%). These estimates reflect the operation of the management protocol rather than independent diagnostic accuracy, because threshold crossing triggered DPC placement and could therefore modify subsequent ischemic risk.

Of the 38 patients who crossed the protocol-defined threshold and underwent selective DPC placement, 36 did not subsequently develop clinically overt limb ischemia.

### 3.5. Mortality and Other Clinical Outcomes

In-hospital mortality was 47.0% (39/83) under the NIRS-guided selective strategy and 44.4% (12/27) with routine prophylactic DPC placement (absolute risk difference, +2.5 percentage points; 95% CI, −18.4 to +22.5; *p* = 0.818). Cardiac death occurred in 37.3% and 29.6%, respectively (*p* = 0.466), and a favorable neurological outcome (CPC 1–2 at discharge) was observed in 48.8% and 51.9%, respectively (*p* = 0.782).

CRRT was required in 43.4% of patients in the selective strategy group and 44.4% in the routine prophylactic DPC group (*p* = 0.922). Sepsis occurred in 27.7% and 33.3% (*p* = 0.576), respectively, and ischemic stroke in 8.4% and 3.7% (*p* = 0.677). Median intensive care unit stay was 15 (8–22) versus 17 (10–28) days (*p* = 0.205), and median hospital stay was 18 (10–58) versus 21 (11–67) days (*p* = 0.352) (Table 2).

### 3.6. Exploratory Subgroup Analyses

Baseline characteristics and outcomes according to eventual DPC status (no DPC, selective DPC, and routine prophylactic DPC) are shown in Tables S2 and S3.

Among patients who underwent ECPR (*n* = 71), in-hospital mortality was 57.1% (16/28) in the no-DPC subgroup, 51.7% (15/29) in the selective DPC subgroup, and 42.9% (6/14) in the routine prophylactic DPC group. Among patients who did not undergo ECPR (*n* = 39), the corresponding mortality rates were 29.4% (5/17), 33.3% (3/9), and 46.2% (6/13), respectively (Figure S1). These subgroup analyses were exploratory and were not powered for formal between-group comparisons.

## 4. Discussion

This study demonstrates the feasibility of a NIRS-guided selective distal perfusion strategy using a single absolute rSO_2_ threshold in an ECPR-dominant VA-ECMO population. Just over half of the patients managed under the selective strategy (54.2%) did not receive a DPC during ECMO support, and no clinically overt limb ischemia was observed among these patients (0/45; exact 95% CI, 0–7.9%). Limb ischemia occurred in 2.4% of patients managed under the selective strategy and in 7.4% of those receiving routine prophylactic DPC; however, the small number of events and nonrandomized treatment allocation preclude conclusions regarding comparative effectiveness.

The rationale for a selective approach is that DPC insertion carries its own risks, including insertion-site bleeding, catheter thrombosis, vascular injury, and pseudoaneurysm formation, which are particularly relevant in critically ill patients receiving anticoagulation. In the present cohort, however, cannula-, vascular-, or circuit-related complications were not less frequent under the selective strategy (14.5% vs. 7.4%). Avoiding a DPC therefore cannot be assumed to reduce access-related complications on the basis of these data. Rather, the principal feasibility finding of this study is that an additional invasive procedure could be avoided in a substantial proportion of patients, with no clinically overt limb ischemia observed among those managed without a DPC.

No clinically overt limb ischemia was observed among the 45 patients managed without a DPC; however, the upper limit of the exact 95% confidence interval was 7.9%, and a clinically relevant residual risk cannot be excluded. This observation supports further evaluation of the protocol but does not establish the safety of deferring DPC placement. Both limb ischemia events under the selective strategy occurred in patients whose rSO_2_ had fallen below the protocol-defined threshold and who had subsequently received a DPC. Conversely, two patients in the routine prophylactic DPC group developed limb ischemia despite DPC placement at cannulation. These observations illustrate that DPC placement does not eliminate limb ischemia risk and suggest that patient-specific vascular factors may remain important irrespective of distal perfusion strategy, although the small number of events precludes firm conclusions.

The comparison with the concurrent routine prophylactic DPC group should be interpreted cautiously. Distal perfusion strategy was selected according to operator preference rather than random allocation, introducing the possibility of confounding by indication and other unmeasured treatment-selection factors. Fluoroscopic guidance was more frequently used in the routine prophylactic DPC group (66.7% vs. 28.9%; SMD, −0.82), and ECMO support duration was longer in that group (median, 8 vs. 6 days). Bilateral NIRS monitoring was performed in both groups, and both strategies were used throughout the study period; these shared features do not eliminate treatment-selection bias. With only 27 patients in the comparator group, nonrandomized allocation based on operator preference, baseline imbalances, and four limb ischemia events overall, the routine prophylactic DPC group should be regarded as an exploratory comparator. These data cannot establish comparative effectiveness or safety, including superiority, equivalence, or noninferiority, between the two strategies.

Our protocol deliberately used a single absolute rSO_2_ threshold from the cannulated limb rather than a relative change from baseline or an inter-limb difference. This approach reflects the clinical context in which the protocol was applied. Most patients underwent cannulation during resuscitation or profound cardiogenic shock, making a reliable pre-cannulation baseline difficult to obtain. Moreover, systemic hypoperfusion and high-dose vasopressor therapy may lower tissue oxygenation in both limbs, limiting the contralateral leg as an internal reference. An absolute threshold therefore provides a simple and reproducible bedside trigger that can be applied during emergency cannulation without requiring a baseline measurement or calculation of an inter-limb difference.

The trade-off for this simplicity was a high rate of DPC placement among patients who did not subsequently develop clinically overt limb ischemia. Of the 38 patients who crossed the threshold and underwent selective DPC placement, 36 did not subsequently develop clinically overt limb ischemia. Whether DPC placement prevented progression to overt ischemia in these patients or represented treatment of desaturation that would not have progressed cannot be determined, because intervention followed the rSO_2_ signal. Consequently, the observed test characteristics of the threshold should be interpreted within the context of this management protocol rather than as independent measures of diagnostic accuracy. Incorporating an inter-limb difference or relative-change criterion, as used in previous NIRS studies, may improve patient selection and represents an important question for prospective evaluation.

Wong et al. used an absolute NIRS threshold of approximately 40% or a relative decline from baseline to identify lower-extremity hypoperfusion during ECMO, while an earlier study from our institution demonstrated that NIRS could detect limb ischemia earlier than conventional clinical assessment [5,15]. Vinogradsky et al. subsequently evaluated 490 patients across pre-NIRS and NIRS eras and reported a reduction in limb ischemia requiring surgical intervention from 8.5% to 2.6% after implementation of continuous NIRS-guided management, although smaller arterial cannulae were also used during the NIRS era; DPC utilization remained similar between eras [18]. More recent studies by Buda et al. and Sohn and Lee have further supported selective DPC management and NIRS-based limb surveillance [4,17].

Earlier selective distal perfusion approaches used clinical examination and Doppler assessment to identify impaired limb perfusion [6,7]. Subsequent studies incorporated continuous NIRS surveillance, using absolute rSO_2_ thresholds, changes from baseline, or inter-limb differences to guide management [5,15,18]. Continuous NIRS monitoring and the use of an absolute threshold are therefore not unique to this study. Our contribution is the evaluation of a predefined single absolute rSO_2_ trigger that does not require baseline or inter-limb reference values, with DPC avoidance explicitly assessed as the primary feasibility endpoint in a predominantly ECPR population. This extends the clinical experience with selective distal perfusion without establishing superiority over other strategies.

Taken together, our findings support further evaluation of monitored, individualized distal perfusion management rather than universal prophylactic DPC placement in femoral VA-ECMO. Continuous NIRS may be particularly useful in sedated or comatose patients who cannot report ischemic symptoms, providing an objective and continuously available physiological signal [19]. A single absolute threshold has the practical advantage of not requiring a pre-cannulation baseline or inter-limb calculation, although whether this simplicity provides the optimal balance between limb protection and additional DPC placement remains uncertain.

### Limitations

This study has several limitations. First, the retrospective, single-center design introduces the possibility of selection bias and limits generalizability. Second, the comparator group was relatively small and non-randomized, and the choice between the NIRS-guided selective strategy and routine prophylactic DPC placement was based on operator preference; confounding by indication and other unmeasured treatment-selection factors therefore cannot be excluded.

Third, only four clinically overt limb ischemia events occurred in the entire cohort. Consequently, the study was not powered to establish superiority, equivalence, or noninferiority between distal perfusion strategies, and the corresponding effect estimates have wide confidence intervals.

Fourth, detailed serial NIRS data, including the nadir rSO_2_, contralateral limb values, and the interval between threshold crossing and DPC insertion, were not systematically captured in the registry. Because no inter-limb criterion was incorporated into the treatment algorithm and contralateral values were unavailable for retrospective analysis, systemic hypoperfusion could not always be distinguished from regional hypoperfusion of the cannulated limb. This limitation may partly explain the low positive predictive value of the absolute rSO_2_ threshold. For the same reason, the proportion of patients in the routine prophylactic DPC group who would have met the trigger, and in whom the catheter was therefore arguably unnecessary, could not be estimated.

Fifth, the registry did not systematically capture the temporal sequence between rSO_2_ decline and the first clinical signs of limb ischemia for each selective DPC placement. Therefore, although clinically overt ischemic events were observed in patients whose rSO_2_ had fallen below the protocol-defined threshold, the precise temporal relationship between physiological desaturation, clinical findings, and DPC insertion could not be reconstructed in all patients.

Sixth, limb ischemia and other vascular complications were ascertained retrospectively from physician-entered medical records. Subclinical or transient ischemic episodes may therefore have been missed, particularly in sedated or comatose patients, although objective findings such as limb color and temperature changes, Doppler signals, compartment syndrome, and therapeutic interventions were used to define clinically overt events.

Seventh, patients who died early had limited exposure time in which to reach the trigger or to develop limb ischemia. In a post hoc analysis excluding the 6 patients who died within 48 h of ECMO initiation, 40 of 77 patients (51.9%; exact 95% CI, 40.3–63.5%) were still managed without a DPC, and no limb ischemia occurred among them.

Several potentially important determinants of limb ischemia were not available in sufficient detail, including peripheral arterial disease severity, femoral artery diameter, cannula-to-vessel ratio, and vasopressor exposure over time. Although the vasoactive-inotropic score at cannulation was recorded, it does not characterize exposure throughout ECMO support. These incompletely measured vascular and hemodynamic factors may have influenced both DPC selection and subsequent limb ischemia risk, leaving residual confounding. SOFA scores were available in only 66 patients, limiting their use for comprehensive risk adjustment, and long-term vascular and functional limb outcomes after hospital discharge were not assessed.

## 5. Conclusions

A NIRS-guided selective distal perfusion strategy based on a single absolute rSO_2_ threshold was feasible in adults supported with femoral VA-ECMO, most of whom underwent cannulation during resuscitation. Just over half of the patients were managed without a distal perfusion catheter, and no clinically overt limb ischemia was observed among them (0/45; exact 95% CI, 0–7.9%). The observed incidence of limb ischemia was low under the selective strategy.

These findings are hypothesis-generating. Prospective, adequately powered studies incorporating serial rSO_2_ trajectories and contralateral limb measurements are needed to assess the safety of deferring prophylactic DPC placement and to determine which criteria best balance limb protection with avoidance of additional invasive catheter placement.

## Figures and Tables

**Table 1:** Baseline and ECMO characteristics according to distal perfusion strategy.

Characteristic	NIRS-Guided Selective DPC (*n* = 83)	Routine Prophylactic DPC (*n* = 27)	*p* Value	SMD
Demographics				
Age, years	66.0 (52.0–74.5)	65.0 (53.5–75.0)	0.602	−0.20
Male sex, *n* (%)	58 (69.9)	20 (74.1)	0.677	−0.09
Height, cm	166.0 (158.5–171.8)	168.0 (161.0–171.5)	0.483	−0.18
Weight, kg	63.0 (55.0–69.9)	65.9 (56.2–75.8)	0.348	−0.20
Comorbidities, *n* (%)				
Hypertension	41 (49.4)	17 (63.0)	0.220	−0.28
Diabetes mellitus	28 (33.7)	10 (37.0)	0.754	−0.07
Smoking, current or former	31 (37.3)	12 (44.4)	0.512	−0.14
Previous myocardial infarction	14 (16.9)	5 (18.5)	1.000	−0.04
Previous PCI	17 (20.5)	5 (18.5)	0.825	0.05
Previous CABG	4 (4.8)	3 (11.1)	0.359	−0.23
Previous cerebrovascular accident	5 (6.0)	3 (11.1)	0.403	−0.18
Current malignancy	6 (7.2)	2 (7.4)	1.000	−0.01
Peripheral artery disease	0 (0.0)	1 (3.7)	0.245	−0.28
Shock severity and ECMO characteristics				
ECPR, *n* (%)	57 (68.7)	14 (51.9)	0.112	0.35
SOFA score	10.0 (8.0–13.0)	9.5 (6.8–12.0)	0.227	0.40
Lactate at shock diagnosis, mmol/L	8.3 (5.4–13.2)	8.4 (4.4–9.7)	0.192	0.21
LVEF before ECMO, %	25.0 (19.0–36.0)	22.0 (17.2–32.9)	0.287	0.20
Vasoactive-inotropic score at cannulation	28.5 (3.0–64.1)	22.9 (12.5–42.5)	0.890	0.03
Fluoroscopic guidance, *n* (%)	24 (28.9)	18 (66.7)	<0.001	−0.82
Arterial cannula 15/17/19 Fr, *n*	54/23/2	19/6/1	0.677	0.15
Initial pump flow, L/min	3.1 (2.8–3.5)	3.0 (2.5–3.5)	0.301	0.16
Unfractionated heparin, *n* (%)	77 (92.8)	27 (100.0)	0.333	−0.39
ECMO support duration, days	6 (3–10)	8 (6–16)	0.024	−0.35

Continuous variables are median (interquartile range) and were compared using the Mann–Whitney U test; categorical variables are *n* (%) and were compared using the chi-square or Fisher’s exact test (arterial cannula size, Fisher–Freeman–Halton exact test). Data were available for the following numbers of patients (selective/routine): SOFA score, 46/20; lactate, 82/25; LVEF, 74/26; vasoactive-inotropic score, 81/27; initial pump flow, 82/27; arterial cannula size, 79/26; ECMO support duration, 82/26. CABG, coronary artery bypass grafting; DPC, distal perfusion catheter; ECMO, extracorporeal membrane oxygenation; ECPR, extracorporeal cardiopulmonary resuscitation; LVEF, left ventricular ejection fraction; NIRS, near-infrared spectroscopy; PCI, percutaneous coronary intervention; SMD, standardized mean difference; SOFA, Sequential Organ Failure Assessment.

**Table 2:** Clinical outcomes according to distal perfusion strategy.

Outcome	NIRS-Guided Selective DPC (*n* = 83)	Routine Prophylactic DPC (*n* = 27)	Risk Difference, Percentage Points (95% CI)	*p* Value
Primary safety outcome, *n* (%)				
Clinically overt limb ischemia	2 (2.4)	2 (7.4)	−5.0 (−21.1 to +3.0)	0.252
ECMO-related complications, *n* (%)				
Cannula-, vascular-, or circuit-related	12 (14.5)	2 (7.4)	+7.1 (−10.0 to +17.6)	0.511
Ischemic stroke	7 (8.4)	1 (3.7)	+4.7 (−10.5 to +13.3)	0.677
Intracranial hemorrhage	0 (0.0)	1 (3.7)	−3.7 (−18.3 to +1.7)	0.245
Gastrointestinal bleeding	4 (4.8)	3 (11.1)	−6.3 (−23.5 to +3.7)	0.359
Major bleeding, non-gastrointestinal	11 (13.3)	1 (3.7)	+9.5 (−6.1 to +19.0)	0.287
Clinical outcomes, *n* (%)				
In-hospital mortality	39 (47.0)	12 (44.4)	+2.5 (−18.4 to +22.5)	0.818
In-hospital cardiac death	31 (37.3)	8 (29.6)	+7.7 (−13.4 to +25.2)	0.466
Favorable CPC (1–2) at discharge	40/82 (48.8)	14/27 (51.9)	−3.1 (−23.4 to +17.7)	0.782
CRRT requirement	36 (43.4)	12 (44.4)	−1.1 (−21.9 to +18.9)	0.922
Dialysis at discharge	13 (15.7)	4 (14.8)	+0.8 (−17.9 to +13.7)	1.000
Sepsis	23 (27.7)	9 (33.3)	−5.6 (−26.3 to +12.4)	0.576
Length of stay, days				
Intensive care unit stay	15 (8–22)	17 (10–28)	—	0.205
Hospital stay	18 (10–58)	21 (11–67)	—	0.352

Risk differences are the selective strategy minus the routine prophylactic DPC group, with Newcombe hybrid score 95% confidence intervals. Length of stay is median (interquartile range) and was compared using the Mann–Whitney U test; all other variables are *n* (%) and were compared using the chi-square or Fisher’s exact test. Favorable neurological outcome was evaluable in 109 patients; one patient in the selective strategy group was transferred before discharge. —, not applicable; risk differences were not calculated for continuous outcomes. CI, confidence interval; CPC, Cerebral Performance Category; CRRT, continuous renal replacement therapy; DPC, distal perfusion catheter; ECMO, extracorporeal membrane oxygenation; NIRS, near-infrared spectroscopy.

## Data Availability

The data presented in this study are available on reasonable request from the corresponding author. The data are not publicly available because they contain information that could compromise the privacy of the patients included in the institutional ECMO registry.

## Supplementary Materials

The following supporting information can be downloaded at: https://www.mdpi.com/article/10.3390/diagnostics16203361/s1, Table S1: Individual characteristics of the four patients with clinically overt limb ischemia; Table S2: Baseline and ECMO characteristics according to eventual distal perfusion catheter status; Table S3: Clinical outcomes according to eventual distal perfusion catheter status; Figure S1: In-hospital mortality according to extracorporeal cardiopulmonary resuscitation (ECPR) status and eventual distal perfusion catheter status. Numerators and denominators are shown within each bar. This exploratory analysis was not powered for formal between-group comparison. DPC, distal perfusion catheter.

## Abbreviations

CPC	Cerebral Performance Category
CRRT	continuous renal replacement therapy
DPC	distal perfusion catheter
ECMO	extracorporeal membrane oxygenation
ECPR	extracorporeal cardiopulmonary resuscitation
IQR	interquartile range
LVEF	left ventricular ejection fraction
NIRS	near-infrared spectroscopy
rSO_2_	regional tissue oxygen saturation
SMD	standardized mean difference
SOFA	Sequential Organ Failure Assessment
VA-ECMO	venoarterial extracorporeal membrane oxygenation
