# Supplementary material for: Near-Infrared Spectroscopy-Guided Selective Distal Perfusion in Femoral Venoarterial Extracorporeal Membrane Oxygenation: A Feasibility Study
Source: Diagnostics (Basel). 2026 Oct 15;16(20):3361. doi: 10.3390/diagnostics16203361 (PMC13703524; doi:10.3390/diagnostics16203361)
Supplement: Supplementary file 1 [file diagnostics-16-03361-s001.zip › diagnostics-4595417-supplementary.pdf]

Supplementary Materials

Near-Infrared Spectroscopy–Guided Selective Distal Perfusion in Femoral Venoarterial Extracorporeal Membrane Oxygenation: A Feasibility Study

Table S1. Individual characteristics of the four patients with clinically overt limb ischemia.

Case	Strategy	Age, Sex; ECPR; Arterial Cannula	Clinical Context	rSO_2 at DPC Trigger	Management	Outcome
1	NIRS-guided selective	41, F; non-ECPR; 15 Fr	Cardiogenic shock initially evaluated for myocarditis, subsequently attributed to pheochromocytoma	<40%	Fasciotomy	Weaned on day 5; survived, CPC 1
2	NIRS-guided selective	40, F; ECPR; 15 Fr	Takayasu arteritis with previous CABG and previous ECMO support; ST-elevation myocardial infarction with cardiac arrest	34%	Emergency fasciotomy for compartment syndrome	Weaned on day 14; survived, CPC 1
3	Routine prophylactic	86, M; ECPR; 15 Fr	Valve coaptation failure during transcatheter aortic valve implantation; left main PCI 2 days later	Not applicable (DPC at cannulation)	Not recorded	In-hospital cardiac death, day 22
4	Routine prophylactic	81, M; non-ECPR; 15 Fr	ST-elevation myocardial infarction with three-vessel disease; ECMO after PCI, subsequent CABG and septic shock	Not applicable (DPC at cannulation)	Not recorded	Weaned on day 8; in-hospital cardiac death, day 12

Day counts run from ECMO initiation (weaning) or from hospital admission (death). rSO_2 values are those recorded at the time the distal perfusion catheter was placed; the interval from cannulation to the ischemic event and the management of the two events in the routine prophylactic DPC group were not recorded in the registry. CABG, coronary artery bypass grafting; CPC, Cerebral Performance Category; DPC, distal perfusion catheter; ECMO, extracorporeal membrane oxygenation; ECPR, extracorporeal cardiopulmonary resuscitation; PCI, percutaneous coronary intervention; rSO_2, regional tissue oxygen saturation.

Table S2. Baseline and ECMO characteristics according to eventual distal perfusion catheter status.

Characteristic	No DPC (n = 45)	Selective DPC (n = 38)	Routine prophylactic DPC (n = 27)	*p* Value
Demographics				
Age, years	68.0 (53.0–75.0)	64.5 (49.8–73.2)	65.0 (53.5–75.0)	0.559
Male sex, n (%)	34 (75.6)	24 (63.2)	20 (74.1)	0.426
Height, cm	167.7 (160.0–171.0)	165.5 (157.0–171.9)	168.0 (161.0–171.5)	0.673
Weight, kg	65.0 (55.2–69.2)	62.0 (53.7–71.3)	65.9 (56.2–75.8)	0.571
Comorbidities, n (%)				
Hypertension	27 (60.0)	14 (36.8)	17 (63.0)	0.051
Diabetes mellitus	18 (40.0)	10 (26.3)	10 (37.0)	0.406
Smoking, current or former	19 (42.2)	12 (31.6)	12 (44.4)	0.494
Previous myocardial infarction	5 (11.1)	9 (23.7)	5 (18.5)	0.327
Previous PCI	6 (13.3)	11 (28.9)	5 (18.5)	0.203
Previous CABG	3 (6.7)	1 (2.6)	3 (11.1)	0.419
Previous cerebrovascular accident	3 (6.7)	2 (5.3)	3 (11.1)	0.725
Current malignancy	1 (2.2)	5 (13.2)	2 (7.4)	0.144
Peripheral artery disease	0 (0.0)	0 (0.0)	1 (3.7)	0.245
Shock severity and ECMO characteristics				
ECPR, n (%)	28 (62.2)	29 (76.3)	14 (51.9)	0.116
SOFA score	10.0 (8.0–12.8)	10.0 (9.0–13.0)	9.5 (6.8–12.0)	0.422
Lactate at shock diagnosis, mmol/L	7.8 (5.4–12.6)	10.8 (5.5–13.9)	8.4 (4.4–9.7)	0.320
LVEF before ECMO, %	25.0 (19.0–38.0)	25.0 (19.0–35.6)	22.0 (17.2–32.9)	0.564
Vasoactive-inotropic score at cannulation	26.0 (7.4–54.0)	35.4 (3.0–80.1)	22.9 (12.5–42.5)	0.776
Fluoroscopic guidance, n (%)	16 (35.6)	8 (21.1)	18 (66.7)	<0.001
Arterial cannula 15/17/19 Fr, n	29/11/2	25/12/0	19/6/1	0.722
Initial pump flow, L/min	3.0 (2.4–3.5)	3.2 (3.0–3.5)	3.0 (2.5–3.5)	0.188

Characteristic	No DPC (n = 45)	Selective DPC (n = 38)	Routine prophylactic DPC (n = 27)	*p* Value
Unfractionated heparin, n (%)	42 (93.3)	35 (92.1)	27 (100.0)	0.433
ECMO support duration, days	6 (3–8)	5 (4–12)	8 (6–16)	0.076

Three-group version of Table 1. The NIRS-guided selective strategy cohort is divided into patients who did not receive a distal perfusion catheter and those who underwent selective placement; subgroup membership was determined by the post-cannulation rSO_2 course rather than assigned at ECMO initiation, so these comparisons are descriptive. Continuous variables were compared using the Kruskal–Wallis test and categorical variables using the chi-square or Fisher–Freeman–Halton exact test (arterial cannula size, Fisher–Freeman–Halton exact test). Data were available for the following numbers of patients (no DPC/selective DPC/routine DPC): SOFA score, 26/20/20; lactate, 44/38/25; LVEF, 41/33/26; vasoactive-inotropic score, 43/38/27; initial pump flow, 45/37/27; arterial cannula size, 42/37/26; ECMO support duration, 45/37/26. DPC, distal perfusion catheter; ECMO, extracorporeal membrane oxygenation; ECPR, extracorporeal cardiopulmonary resuscitation; LVEF, left ventricular ejection fraction; NIRS, near-infrared spectroscopy; PCI, percutaneous coronary intervention; rSO_2, regional tissue oxygen saturation; SOFA, Sequential Organ Failure Assessment.

Table S3. Clinical outcomes according to eventual distal perfusion catheter status.

Outcome	No DPC (n = 45)	Selective DPC (n = 38)	Routine prophylactic DPC (n = 27)	*p* Value
Primary safety outcome, n (%)				
Clinically overt limb ischemia	0 (0.0)	2 (5.3)	2 (7.4)	0.166
ECMO-related complications, n (%)				
Cannula-, vascular-, or circuit-related	5 (11.1)	7 (18.4)	2 (7.4)	0.386
Ischemic stroke	4 (8.9)	3 (7.9)	1 (3.7)	0.808
Intracranial hemorrhage	0 (0.0)	0 (0.0)	1 (3.7)	0.245
Gastrointestinal bleeding	2 (4.4)	2 (5.3)	3 (11.1)	0.607
Major bleeding, non-gastrointestinal	5 (11.1)	6 (15.8)	1 (3.7)	0.315
Clinical outcomes, n (%)				
In-hospital mortality	21 (46.7)	18 (47.4)	12 (44.4)	0.972
In-hospital cardiac death	17 (37.8)	14 (36.8)	8 (29.6)	0.764
Favorable CPC (1–2) at discharge	23/45 (51.1)	17/37 (45.9)	14/27 (51.9)	0.864
CRRT requirement	15 (33.3)	21 (55.3)	12 (44.4)	0.133
Dialysis at discharge	6 (13.3)	7 (18.4)	4 (14.8)	0.799
Sepsis	12 (26.7)	11 (28.9)	9 (33.3)	0.834
Length of stay, days				
Intensive care unit stay	13 (7–20)	16 (8–30)	17 (10–28)	0.238
Hospital stay	17 (10–53)	18 (12–70)	21 (11–67)	0.457

Three-group version of Table 2. Favorable neurological outcome was evaluable in 45/37/27 patients (no DPC/selective DPC/routine prophylactic DPC); one patient in the selective DPC subgroup was transferred before discharge. CPC, Cerebral Performance Category; CRRT, continuous renal replacement therapy; DPC, distal perfusion catheter; ECMO, extracorporeal membrane oxygenation.

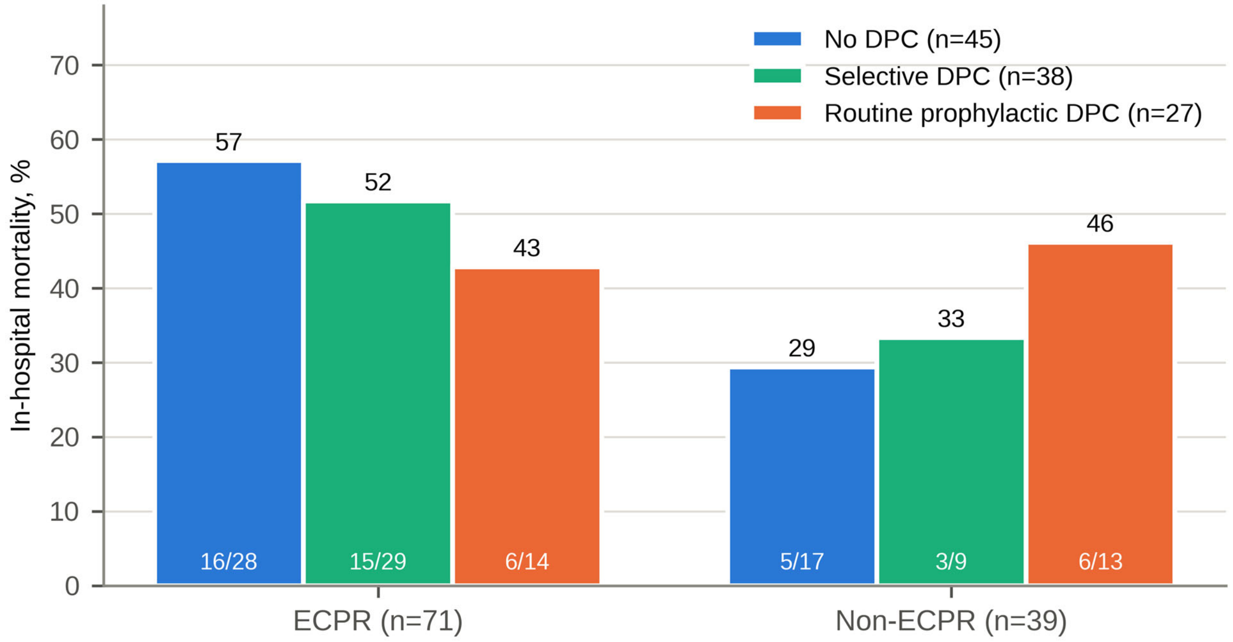

Figure S1. In-hospital mortality according to extracorporeal cardiopulmonary resuscitation (ECPR) status and eventual distal pe rfusion catheter status. Numerators and denominators are shown within each bar. This exploratory analysis was not powered fo r formal between-group comparison. DPC, distal perfusion catheter.
